# Soy-isoflavone to remedy induced or post-gamma-irradiation periodontitis in rat model

**DOI:** 10.1186/s12903-026-08604-z

**Published:** 2026-05-29

**Authors:** Dina M. Hegab, Mostafa A. Bakr, Amr H. Rasmy, Ahmed Abdelrahman, Amira I. Sayed

**Affiliations:** Health Radiation Research Dept, National Center for Radiation Research and Technology, Egyptian Atomic Energy Authority, Cairo, Egypt

**Keywords:** Isoflavone, Periodontitis, Gamma radiation, Ligature model, Rat, CBCT

## Abstract

**Background:**

Radiotherapy remains a cornerstone in tumour management; however, it can lead to deleterious effects on alveolar bone, including hypovascularity, endarteritis, and osteoradionecrosis. Periodontitis is a chronic inflammatory disease characterized by progressive alveolar bone loss and eventual tooth loss. Isoflavones, plant-derived phytoestrogens, have been recognized for their bone-protective and anti-osteoporotic properties. This study aimed to investigate the therapeutic efficacy of two different concentrations of isoflavones in mitigating gamma radiation– and ligature-induced periodontitis in rats’ mandibles.

**Methods:**

Forty-eight male albino rats were divided into six groups: gamma radiation-induced periodontitis (R), ligature-induced periodontitis (P), gamma radiation-induced periodontitis treated with 20 mg/kg isoflavones (R20), ligature-induced periodontitis treated with isoflavones at 20 mg/kg (P20), gamma radiation-induced periodontitis treated with 50 mg/kg isoflavones (R50), and ligature-induced periodontitis treated with isoflavones at 50 mg/kg (P50). Following euthanasia, mandibles were divided into two halves for radiographic bone density and histopathological assessments.

**Results:**

The lowest bone density values were recorded in the R group, while the P50 group exhibited the highest bone density. A trend toward improvement in bone mineralization with increasing dose of isoflavones. Histologically, the R group showed hypovascular Haversian canals, osteocytes with eccentric nuclei, and enlarged lacunae. R20 group demonstrated partially restored vascularity, Haversian canals containing blood vessels, and osteocytes showing wide and narrow lacunae. R50 group exhibited marked improved histoarchitecture, narrow Haversian canals containing red blood cell-filled vessels, and well-organized bone matrix. In the P group, marked alveolar bone and periodontal ligament loss were evident, with osteocytes exhibiting enlarged lacunae. Isoflavone at 20 mg/kg (P20) resulted in partial regeneration of periodontal fibers and normalization of lacunar morphology, whereas a 50 mg/kg dose (P50) led to pronounced periodontal regeneration, narrow Haversian canals containing vascular structures, and distinct Volkmann’s, Zuckerkandl, and Hirschfeld canals.

**Conclusion:**

Isoflavones, particularly at a concentration of 50 mg/kg body weight, effectively preserved bone microarchitecture, enhanced vascularization, and promoted periodontal tissue regeneration. These findings suggest that isoflavone may serve as a promising adjunctive therapeutic agent for mitigating radiation-associated and inflammatory alveolar bone loss.

## Background

Radiotherapy (RT) is a cornerstone therapy for managing malignant tumors, administered either as a standalone treatment or in conjunction with surgery and/or chemotherapy [1]. Although patients with early-stage disease generally exhibit favorable survival outcomes following head and neck RT, it can adversely affect adjacent healthy tissues, leading to side effects including xerostomia, dental caries, and osteoradionecrosis [2].

Periodontal disease is a chronic inflammatory infection that leads to the destruction of the tooth-supporting structures, including the gingiva, cementum, periodontal ligaments, and alveolar bone, primarily through connective tissue attachment loss and bone resorption. Its mildest manifestation, gingivitis, is a reversible inflammatory response, whereas periodontitis represents a more severe, chronic, and irreversible form of the disease [3].

Periodontitis is a bacterial-induced chronic inflammatory condition characterized by gingival bleeding, connective tissue detachment, and the formation of periodontal pockets; if left untreated, it can result in progressive alveolar bone destruction and eventual tooth loss [4]. The periodontium is among the tissues most sensitive to radiation. Both the direct and indirect effects of radiation contribute to increased attachment loss, reduced vascularity and cellularity of the periodontal membrane, and diminished blood supply. Additionally, radiation can induce hypovascularity of the alveolar bone, endarteritis, and elevate the risk of osteoradionecrosis [5].

Alternative medicines and natural remedies have attracted increasing research interest due to their affordability and lower incidence of adverse effects [6]. Isoflavones are plant-derived phytoestrogens known for their strong estrogenic activity. The most biologically active forms, genistein, glycitein, and daidzein, are primarily obtained from soybeans. Among these, genistein and daidzein have demonstrated potential roles in the prevention of prostate and breast cancers, as well as osteoporosis [7].

Accordingly, this study was designed to evaluate the therapeutic potential of two different concentrations of isoflavones against gamma radiation-induced and ligature-induced periodontitis in the mandibles of albino rats. The null hypothesis stated that neither isoflavone concentration would play a therapeutic role against gamma radiation-induced or ligature-induced periodontitis in the mandibles of albino rats.

## Methods

**Fig. 1:**
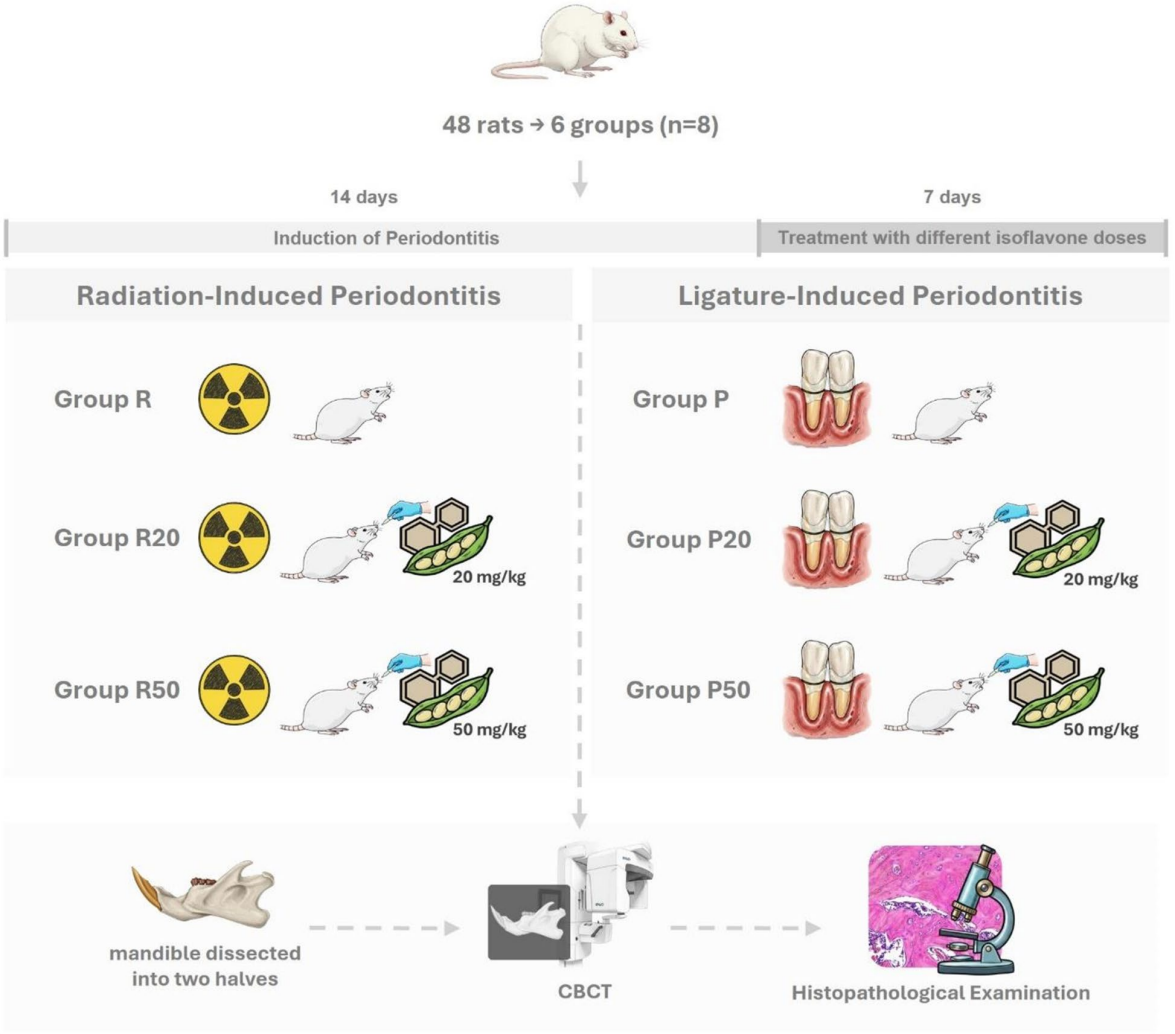
Graphical summary of the experimental workflow. Graphics generated using Gemini 2.5 (Google DeepMind, 2025)

This experimental study was conducted to evaluate the potential therapeutic role of two different concentrations of isoflavones against gamma radiation-induced and ligature-induced periodontitis in the mandibles of albino rats. Bone density analysis and histopathological evaluation methods were employed to evaluate treatment outcomes. The experiment’s workflow is illustrated in Fig. 1.

### Ethics statement and animal care

The experiment was conducted according to the protocol approved by the Research Ethics Committee of the National Center for Radiation Research and Technology (REC-NCRRT), Egyptian Atomic Energy Authority, no. 23 A/ 23.

### Sample size calculation

Based on Liu et al. [8], the sample size was determined using the Bivariate Normal Distribution Model (G power statistical analysis program, Version 3.1.9.4) and using one-way analysis of variance (ANOVA) utilizing a prior power analysis, with values of effect size Cohen f = 0.602, an actual power (1-β error) of 0.8 (80%) and primary risk of error (α = 0.05). A total sample size of 42 was estimated. Taking into consideration the additional 15% probability for potential dropout, the final sample size generated for bone density was 48 rats, subdivided into eight in each group (*n* = 8).

### Animals and grouping

Forty-eight adult male albino rats (190 ± 10 g, approximately 8 weeks old) were obtained from the breeding unit of the National Centre for Radiation Research and Technology (NCRRT), Egyptian Atomic Energy Authority, Cairo, Egypt. The animals were acclimatized to laboratory conditions for two weeks before starting the experiment. They were housed in polypropylene/stainless-steel cages (54 × 37 × 27 cm) under controlled environmental conditions, including adequate ventilation, relative humidity not exceeding 55%, temperature between 25 and 28 °C, and a 12-hour light/dark cycle. Standard pellet diet and drinking water were provided *ad libitum*. Cage bedding was changed, and cages were cleaned daily, with disinfection performed every three days. Animal care was conducted by the staff of the NCRRT animal facility, while the investigators were responsible for performing euthanasia procedures. No humane endpoints were anticipated during the course of the study.

The rats were randomly assigned to six groups (*n* = 8). Group R: gamma radiation-induced periodontitis, without any treatment. Group P: ligature-induced periodontitis without any treatment. Group R20: gamma radiation-induced periodontitis followed by administration of isoflavones (20 mg/kg body weight). Group P20: ligature-induced periodontitis followed by administration of isoflavones (20 mg/kg body weight). Group R50: gamma radiation-induced periodontitis followed by administration of isoflavones (50 mg/kg body weight). Group P50: ligature-induced periodontitis followed by administration of isoflavones (50 mg/kg body weight).

### Gamma-irradiation

Rats of groups R, R20, and R50 were fully immobilized inside a specialized shielding apparatus. They were then exposed to a single localized cranial gamma-irradiation dose of 20 Gy [9] at a dose rate of 0.600 KGy/h. Irradiation was performed using a ^60^Co Gamma Cell (Model 220, India) at the National Centre for Radiation Research and Technology (NCRRT), Egyptian Atomic Energy Authority, Cairo, Egypt. Before irradiation, animals were intra-peritoneally anesthetized with a combination of ketamine (90 mg/kg) and xylazine (10 mg/kg) in a 2:1 ratio (0.12 ml/100 g body weight) as described by Yano et al. [10].

### Induction of periodontitis

Rats assigned to groups P, P20, and P50 were positioned on the surgical table and anesthetized via intraperitoneal injection of a ketamine-xylazine mixture (ketamine 90 mg/kg and xylazine 10 mg/kg, 2:1 ratio; 0.12 ml/100 g body weight) [10]. Subsequently, a 3 − 0 sterile black silk ligature was gently placed around the cervical region of the mandibular incisors using two microsurgical needle holders, with a dental spatula serving as a tongue retractor. The ligatures were stabilized with flowable composite resin to ensure retention and promote periodontitis induction, following the protocol of Tomina et al. [11]. Ligatures were inspected in all animals on day 7 post-ligation. Any animal exhibiting ligature displacement or loss at that time point was excluded from the study.

### Preparation and administration of isoflavone doses

Isoflavone was supplied as a commercial human-use capsule (FlavonPause™, soft gel capsules, SIGNIFY NATURE, Spain, and packaged in the USA). Each capsule contained a total of 124 mg of isoflavones. For the primary stock, one capsule (124 mg) was dispersed in 10 mL of corn oil as a vehicle [12, 13], yielding a concentration of 12.4 mg/mL. Individual doses were calculated according to body weight using the formula:

$$\begin{aligned} & \text{Dose volume (mL)} \\ &= \left(\text{desired dose, mg/kg} \times \text{body weight, kg}\right)\\ & /\text{stock concentration (mg/mL).}\end{aligned}$$

For a 200 g (0.2 kg) rat, this corresponds to approximately 0.32 mL for the 20 mg/kg dose (4 mg ÷ 12.4 mg/mL) and 0.81 mL for the 50 mg/kg dose (10 mg ÷ 12.4 mg/mL). The preparation was freshly mixed before each administration to ensure uniform distribution of the compound within the oil vehicle.

Fourteen days following gamma-irradiation and induction of periodontitis [14], isoflavone was administered to rats once daily for seven consecutive days by oral gavage using a 1-mL (100-unit) insulin syringe fitted to the oral gavage. Two dosage regimens were employed: 20 mg/kg body weight for groups R20 and P20 [11, 14, 15], and 50 mg/kg body weight for groups R50 and P50 [16].

### Euthanasia of animals

At the end of the experimental period (the day following the final isoflavone doses), all rats were humanely euthanized by an overdose of ketamine and xylazine [17]. Each mandible was then carefully dissected into two halves: one-half was allocated for bone density analysis, while the other was processed for histopathological evaluation.

### Bone density analysis

Before CBCT imaging, the mandibles were immersed in formalin to achieve tissue fixation and preservation, maintain bone density stability, and ensure biosafety during image acquisition. Subsequently, the specimens were placed in plastic containers for scanning. These containers were positioned within the CBCT unit using empty polystyrene boxes to achieve the optimal height for tomographic scanning. The obtained tomographic images were analyzed by a blinded examiner using Planmeca Romexis software through the Multi-Planar Reconstruction (MPR) screen, allowing assessment in sagittal, coronal, and axial planes and determination of the region of interest (ROI). Bone density within the periodontitis region, specifically the interdental area between the two central incisors, was measured on sagittal plane images for all ROIs using the point tool, expressed in Hounsfield units (HU). For density measurement, a standardized area of 1.2 mm² was selected to ensure consistency of data collection and enable reliable statistical analysis [18].

### Histopathological evaluation

The anterior regions of the dissected mandibles, including teeth, bone, and surrounding soft tissues, were collected and fixed in 10% phosphate-buffered formalin (pH 7.4) for 24 h. Specimens were then decalcified using a mixed acid solution containing hydrochloric acid and formic acid (4–5%). Following decalcification, samples were dehydrated through a graded alcohol series, cleared in xylene, and embedded in paraffin [19]. Serial sections of 5-µm thickness were prepared and stained with hematoxylin and eosin (H&E). Histopathological evaluation was performed using a Leica Qwin 500 image analysis system (Leica, England) at the Pathology Department, Faculty of Dental Medicine for Girls, Al Azhar University, Cairo, Egypt.

### Statistical analysis

Statistical analysis was done using one-way ANOVA to compare the values of bone density among the experimental groups. Statistical significance was defined as *p* < 0.05. Data were analyzed using the Statistical Package for the Social Sciences (SPSS) software, version 26. The distribution of quantitative variables was assessed for normality using the Shapiro-Wilk and Kolmogorov-Smirnov tests and for homogeneity of variance using the Levene’s test. Normally distributed data were expressed as mean ± standard deviation, while non-normally distributed data were reported as median and range.

## Results

**Fig. 2:**
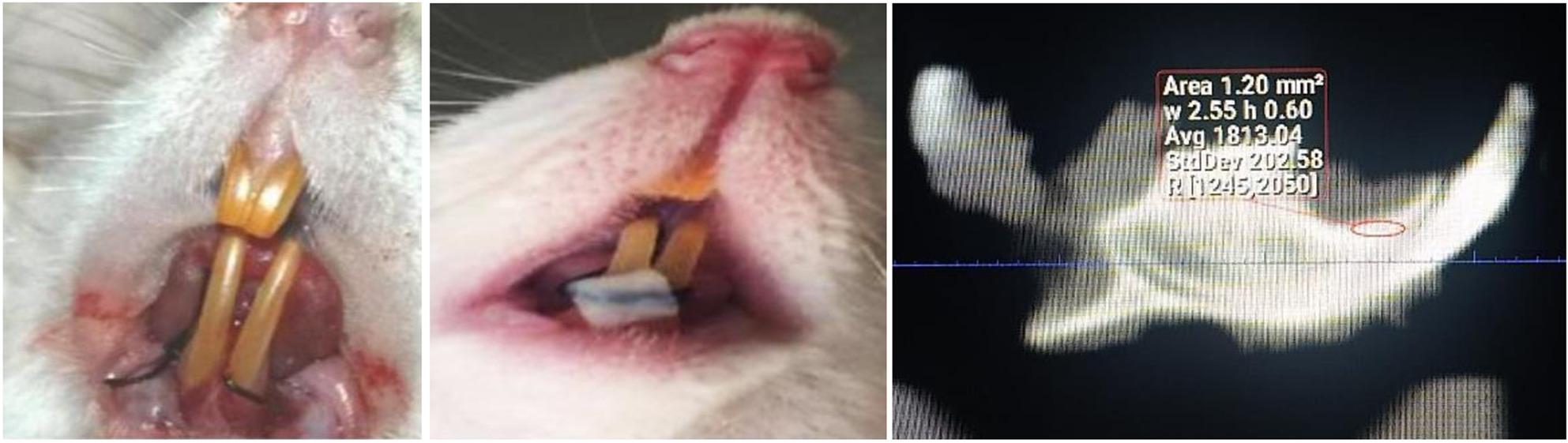
Clinical and radiographic images demonstrating an established periodontitis

The therapeutic potential of two different concentrations of isoflavones was evaluated against gamma radiation-induced and ligature-induced periodontitis in the mandibles of male albino rats. Six experimental groups were analyzed: untreated gamma radiation-induced periodontitis, untreated ligature-induced periodontitis, gamma radiation-induced periodontitis treated with 20 mg/kg body weight isoflavones, ligature-induced periodontitis treated with 20 mg/kg body weight isoflavones, gamma radiation-induced periodontitis treated with 50 mg/kg body weight isoflavones, and ligature-induced periodontitis treated with 50 mg/kg body weight isoflavones. The establishment of periodontitis was validated through clinical and radiographic assessments (Fig. 2). The outcomes were radiographically and histopathologically assessed once at the end of the study.

### Bone density results

**Fig. 3:**
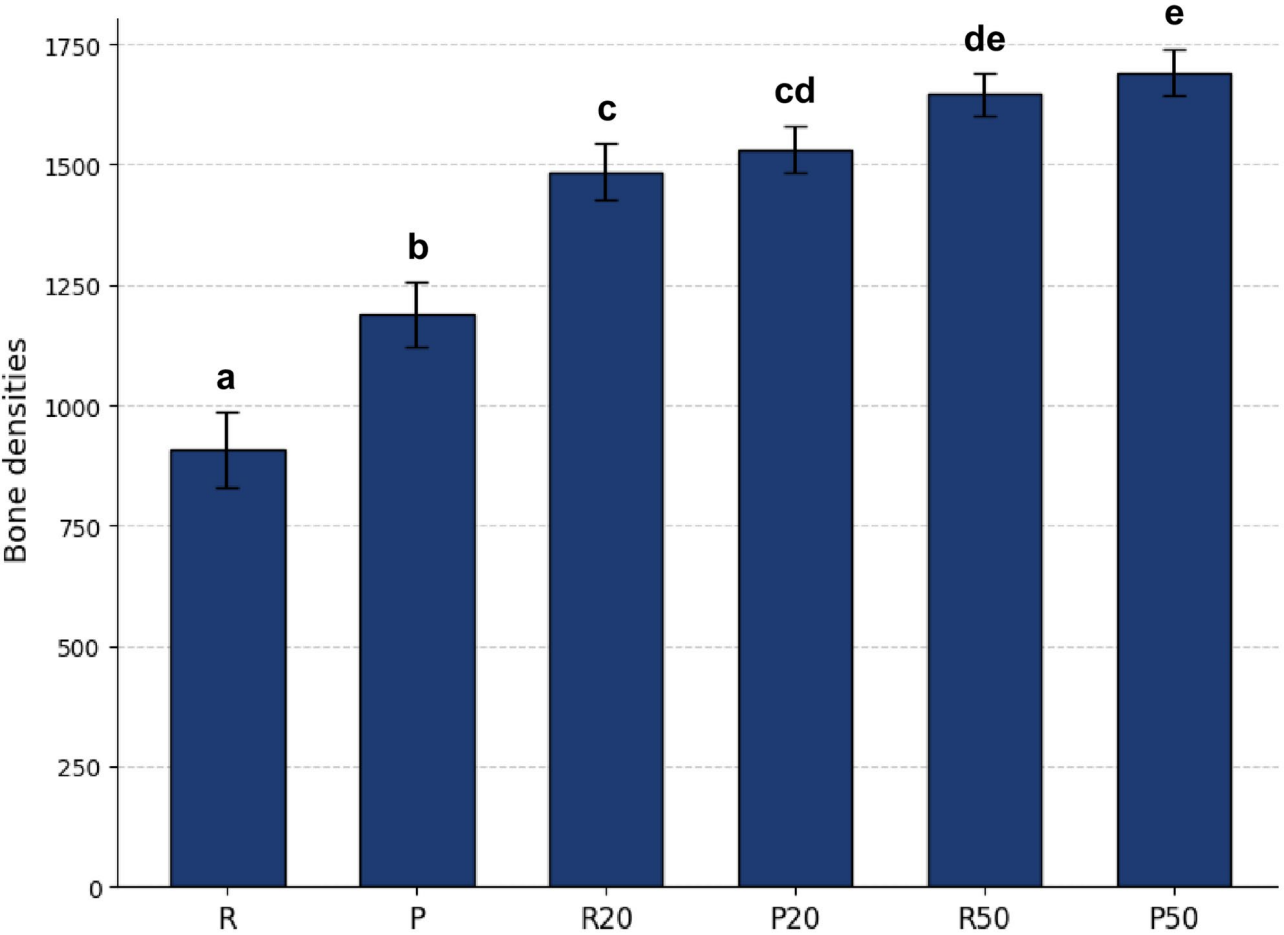
Bar graph represents mean values of bone density of different experimental groups. Different letters mean significant (*P* ≤ 0.05)

The mean bone density values (Mean ± SD) across the different experimental groups were summarized in Table 1; Fig. 3. A one-way analysis of variance (ANOVA) revealed a statistically significant difference in bone density among the experimental groups (*P* < 0.001). The lowest mean bone density was observed in the R group (907.60 ± 77.41), while the highest value was recorded in the P50 group (1690.20 ± 48.11), representing an approximate 86% increase relative to the R group. Progressive increases in bone density were observed with treatment, particularly in groups receiving higher doses (R50 and P50).

**Table 1:** Mean ± standard deviation of bone density values in different experimental groups

Group	Mean & SD	95% Confidence Interval for Mean		Min	Max	F	P value
		Lower Bound	Upper Bound				
R	907.60 ±77.41^a^	811.48	1003.71	807	998	134.99	*P*<0.001*
P	1189 ±66.64^b^	1106.24	1271.75	1117	1266		
R20	1485.20 ±57.31^c^	1414.04	1556.35	1407	1567		
P20	1531 ±47.79^cd^	1471.65	1590.34	1461	1578		
R50	1646.60 ±44.16^de^	1591.76	1701.43	1589	1692		
P50	1690.20 ±48.11^e^	1630.46	1749.93	1639	1746		

Different letters mean significant (*P* ≤ 0.05)

Post hoc multiple comparison testing, using Bonferroni correction, (Table 2) demonstrated that all treated groups showed significantly higher bone density compared with the irradiated untreated group (R) (*P* < 0.001). Similarly, the P group showed significantly reduced bone density compared to all other groups (*P* < 0.001) although it remained significantly higher than that of the R group (*P* < 0.001).

**Table 2:** Pairwise comparisons regarding bone densities using Post hoc analysis

(I) Group	(J) Group	Mean Difference (I-J)	Sig.	95% Confidence Interval	
				Lower Bound	Upper Bound
R	P	-281.40-^*^	.000	-401.17-	-161.62-
	R20	-577.60-^*^	.000	-697.37-	-457.82-
	P20	-623.40-^*^	.000	-743.17-	-503.62-
	R50	-739-^*^	.000	-858.77-	-619.22-
	P50	-782.60-^*^	.000	-902.37-	-662.82-
P	R	281.40^*^	.000	161.62	401.17
	R20	-296.20-^*^	.000	-415.97-	-176.42-
	P20	-342-^*^	.000	-461.77-	-222.22-
	R50	-457.60-^*^	.000	-577.37-	-337.82-
	P50	-501.20-^*^	.000	-620.97-	-381.42-
R20	R	577.60^*^	.000	457.82	697.37
	P	296.20^*^	.000	176.42	415.97
	P20	-45.80-	1.000	-165.57-	73.97
	R50	-161.40-^*^	.003	-281.17-	-41.62-
	P50	-205-^*^	.000	-324.77-	-85.22-
P20	R	623.40^*^	.000	503.62	743.17
	P	342^*^	.000	222.22	461.77
	R20	45.80	1.000	-73.97-	165.57
	R50	-115.60-	.066	-235.37-	4.17
	P50	-159.20-^*^	.003	-278.97-	-39.42-
R50	R	739^*^	.000	619.22	858.77
	P	457.60^*^	.000	337.82	577.37
	R20	161.40^*^	.003	41.62	281.17
	P20	115.60	.066	-4.17-	235.37
	P50	-43.60-	1.000	-163.37-	76.17
P50	R	782.60^*^	.000	662.82	902.37
	P	501.20^*^	.000	381.42	620.97
	R20	205.00^*^	.000	85.22	324.77
	P20	159.20^*^	.003	39.42	278.97
	R50	43.60	1.000	-76.17-	163.37

* means significant at *p* ≤ 0.05

Treated groups (R20, P20, R50, and P50) exhibited significant differences relative to each other in most pairwise comparisons. Both R20 and P20 groups exhibited significantly higher bone density compared to the R and P groups (*P* < 0.001). Moreover, R50 and P50 groups displayed significantly greater bone density in comparison to R, P, and R20 groups (*P* < 0.001). Furthermore, the P50 group showed a significantly higher bone density compared to the P20 group (*P* < 0.001).

No significant difference was detected between R20 and P20 (*P* = 1.000), or between R50 and P50 (*P* = 1.000), indicating comparable effects at the same dose levels. However, increasing the treatment dose of isoflavone from 20 mg/kg to 50 mg/kg resulted in a marked increase in bone density within both irradiated and non-irradiated groups (*P* < 0.01). Overall, the P50 group exhibited the highest bone density values, suggesting a trend toward improvement in bone density with increasing dose following isoflavone treatment.

### Histopathological results

**Fig. 4:**
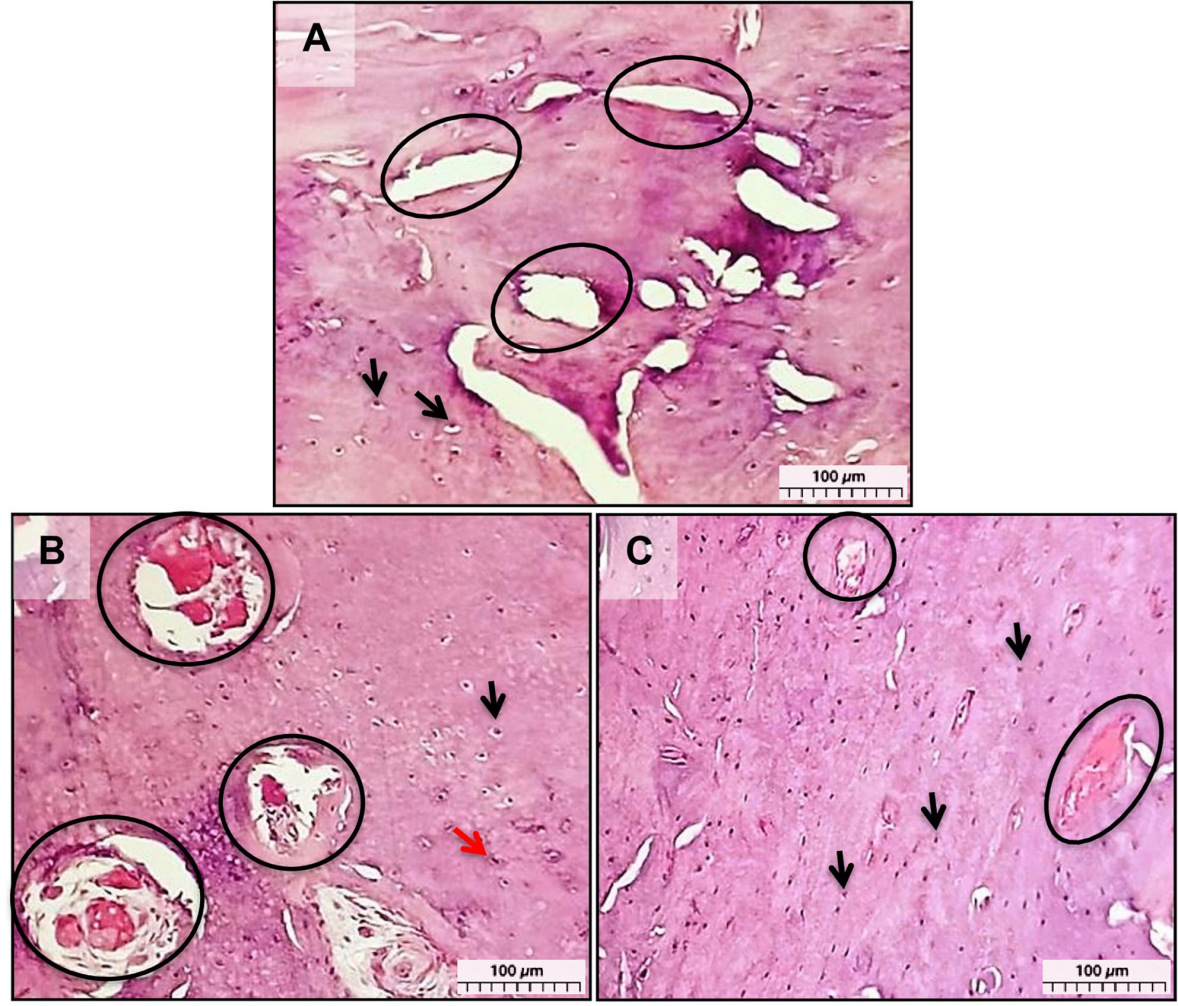
Photomicrographs of H&E stained sections of mandibular bone of rats demonstrating histopathological alterations under different experimental conditions (×100). **A **Group R: Haversian canals (black circles) appeared devoid of blood vessels and osteocytes exhibited wide osteocytic lacunae (black arrows). **B **Group R20: Haversian canals were filled with engorged blood vessels (black circles) and osteocytes showed both wide (black arrows) and narrow (red arrows) osteocytic lacunae. **C **Group R50: Haversian canals (black circles) appeared narrow, and osteocytes displayed narrow osteocytic lacunae (black arrows)

Histological examination revealed qualitative changes in the examined tissues, as observed in H&E stained sections. In group (R), which received localized gamma-irradiation only without treatment, the compact bone exhibited Haversian canals devoid of blood vessels. The osteocytes displayed eccentric nuclei and enlarged osteocytic lacunae (Fig. 4A). Nonetheless, in group (R20), where rats were treated with isoflavone (20 mg/kg body weight) following gamma radiation-induced periodontitis, the Haversian canals appeared engorged with blood vessels. Osteocytes generally exhibited wide lacunae, although some areas showed osteocytes with narrower lacunae (Fig. 4B). In contrast, the R50 group, treated with a higher dose of isoflavones (50 mg/kg body weight) after gamma radiation-induced periodontitis, demonstrated Haversian canals of normal width containing blood vessels filled with red blood cells (RBCs). Osteocytes appeared normally distributed, with narrow lacunae and well-organized architecture (Fig. 4C).

**Fig. 5:**
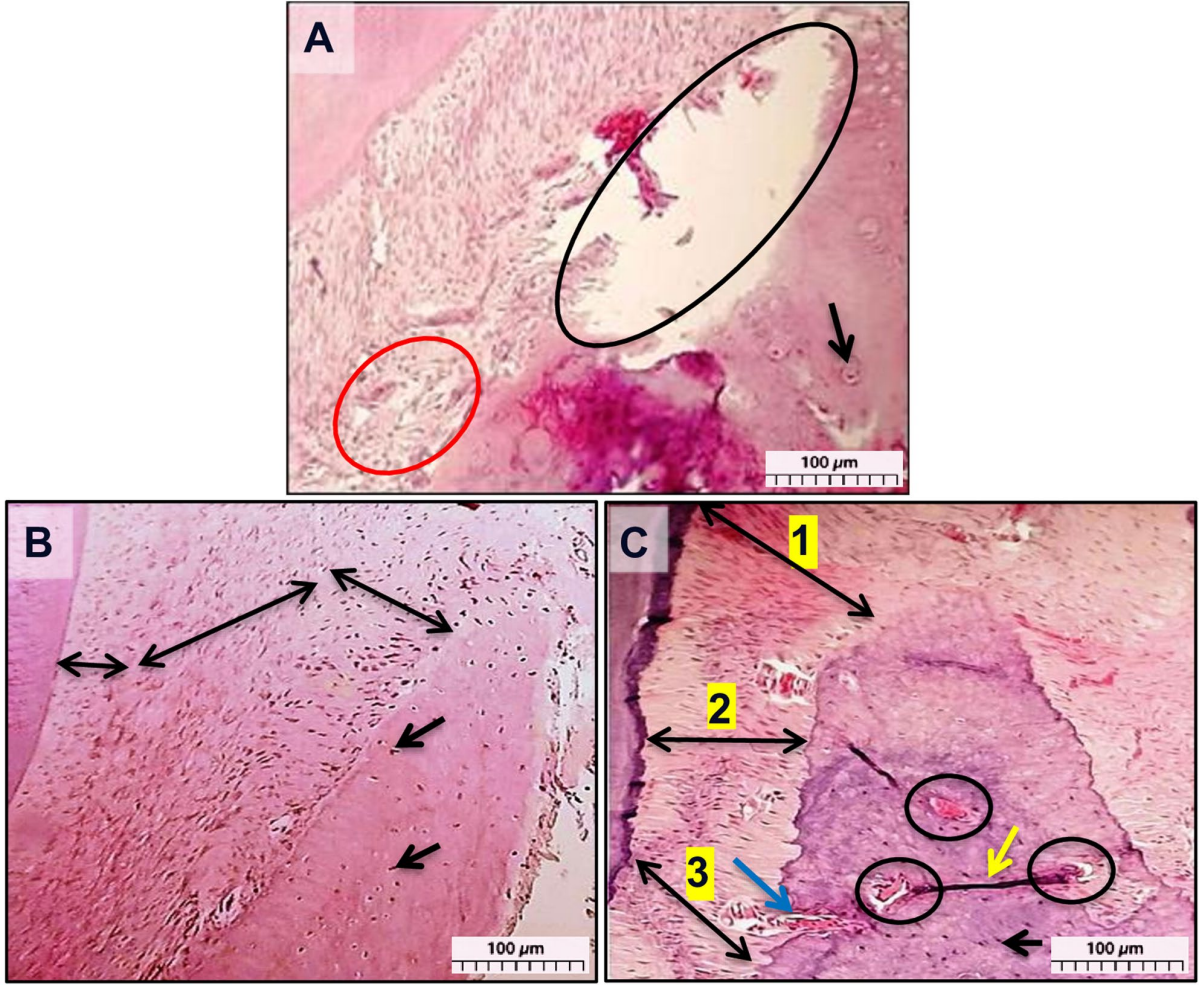
Photomicrographs of H&E stained sections of mandibular bone of rats demonstrating histopathological alterations under different experimental conditions (×100). **A **Group P: Marked loss of bone and attachment (black circle), distortion of periodontal ligament fibers (red circle), and osteocytes exhibiting widened lacunae (black arrows). **B** Group P20: Partial re-establishment of periodontal fibers with altered orientation (black double-headed arrows) and osteocytes showing normal lacunae (black arrows). **C **Group P50: Restoration of periodontal fibers with normal arrangement, including alveolar crest (1), horizontal (2), and oblique (3) fibers. Narrow Haversian canals (black circles), Volkmann’s canal (yellow arrow), and Zuckerkandl and Hirschfeld canals (blue arrow) are evident

In group (P), which was subjected to ligature-induced periodontitis without treatment, marked loss of bone and periodontal ligament attachment was evident. Some areas showed early distortion of the periodontal ligament, while osteocytes exhibited enlarged lacunae (Fig. 5A). However, in group (P20), which received isoflavone treatment (20 mg/kg body weight) following ligature-induced periodontitis, partial re-establishment of periodontal fibers was observed, though their orientation appeared irregular. Osteocytes generally exhibited normal lacunae but with an irregular distribution (Fig. 5B). Nevertheless, in the P50 group, treated with a higher dose of isoflavone (50 mg/kg body weight) following periodontitis induction, marked regeneration of the periodontal fibers was observed with improved alignment of alveolar crest, horizontal, and oblique fibers. The Haversian canals appeared narrow and contained blood vessels filled with RBCs. Additionally, Volkmann’s canals were evident, along with the nutrient canal of Zuckerkandl and Hirschfeld (Fig. 5C).

## Discussion

The current study aimed at investigating the therapeutic effects of two concentrations (20 and 50 mg/kg) of isoflavones on gamma radiation- and ligature-induced periodontitis in adult male albino rats, a model replicating the oral complications commonly observed in head and neck cancer patients receiving radiotherapy (RT). Six experimental groups were established, comprising untreated and isoflavone-treated (20 or 50 mg/kg) models of gamma radiation- and ligature-induced periodontitis.

Head and neck cancers are considered a major global health concern, accounting for more than 890,000 new cases and approximately 450,000 deaths yearly [20]. Despite its therapeutic efficacy, head and neck RT is associated with adverse oral effects, including deterioration of dental and periodontal tissues and an increased risk of developing osteoradionecrosis (ORN) [21]. These adverse manifestations are the result of reactive oxygen species (ROS), which destroy periodontal tissues via activating the expression of nuclear factor-kappa b, releasing pro-inflammatory cytokines, as well as triggering the receptor activator of nuclear factor kappa beta ligand [22].

Periodontitis is a common, chronic inflammatory and microbial disease characterized by the progressive destruction of tooth-supporting tissues [23]. Clinically, it can lead to pain, infection of the bone surrounding dental roots, and eventual tooth loss [4, 24]. In patients with head and neck cancer (HNC), periodontitis may also act as a predisposing factor for ORN. Radiotherapeutic patients exhibit a higher susceptibility to periodontal disease than the general population. Radiation-induced hyposalivation reduces the protective functions of saliva, increasing vulnerability to periodontitis. Additionally, RT alters the composition of the oral microbiome, with a shift in microbial composition and an increase in pathogenic bacterial colonization [25].

Given its clinical relevance, oral periodontitis represents an important experimental model for investigating bone density. Periodontitis does not spontaneously occur in rats; therefore, experimental induction is required to establish a reliable disease model. Commonly used approaches include bacterial inoculation or the placement of ligatures around the teeth. These methods promote plaque accumulation, provoke gingival inflammation, and ultimately lead to alveolar bone resorption, thereby simulating the pathological features of human periodontitis in a controlled and reproducible manner. This model is valuable for investigating novel therapeutic strategies, particularly those targeting the heightened susceptibility to systemic disorders associated with periodontal inflammation [11].

Ligature placement remains one of the most commonly employed methods for experimentally inducing periodontal disease in animals. This approach offers several notable advantages, particularly improved accessibility, especially to the incisor region, and a technically straightforward operative procedure. Together, these features contribute to its widespread use in experimental periodontal research [11]. For short-term investigations, the rat model is particularly advantageous, offering practical benefits related to cost, procedural efficiency, ease of handling, and reduced animal use. Induction of periodontitis by applying a ligature around the rat teeth has been widely described in the literature [26–29].

Periodontitis, whether gamma radiation- or ligature-induced, is marked by progressive alveolar bone resorption, degradation of the periodontal ligament, and deterioration of bone microarchitecture. To the best of our knowledge, this is the first study to investigate the therapeutic efficacy of isoflavone for gamma radiation- and ligature-induced periodontitis in rats. An enhancement in alveolar bone density and overall histological organization was evident, highlighting the role of isoflavone in modulating periodontal tissue integrity under conditions of oxidative stress and chronic inflammation.

Radiographic assessment demonstrated that the gamma-irradiated group (R) exhibited the most pronounced bone loss, reflected by the lowest mean bone density among all groups. The qualitative histopathological analysis supported the radiographic observation, where mandibular sections demonstrated obvious features of radiation-induced bone damage, characterized by empty or dilated Haversian canals, osteocytes with eccentric nuclei, and enlarged lacunae, indicative of cellular degeneration and compromised vascularity. These results align with previous reports describing radiation-associated disruption of bone microcirculation and osteocyte viability [21, 24, 30, 31].

A previous investigation of mandibular changes after radiotherapy for oral cancer demonstrated substantial structural and vascular deterioration. Histologically, cortical and trabecular bone showed pronounced resorption, numerous empty osteocytic lacunae, and mineralized deposits occluding the Haversian and Volkmann canals. Marked vascular compromise and disorganization of elastic fibers were also observed. Radiographically, the mandible displayed irregular osteolytic areas interspersed with prominent osteosclerotic changes [32]. Consistently, one week after irradiation, mice demonstrated significant trabecular bone loss accompanied by elevated osteoclast activity and increased osteocyte apoptosis. Although osteoblast numbers remained unchanged, bone formation was nevertheless impaired, as indicated by a decreased mineralized surface and an accumulation of unmineralized osteoid [33].

Another investigation employing total irradiation doses of 35–45 Gy demonstrated a pronounced reduction in osteocytes, accompanied by an increased prevalence of empty lacunae, reflecting osteocyte loss in irradiated rabbit mandibles [34]. Likewise, histological analysis of rabbits’ mandibles following a single, non-fractionated 30 Gy exposure revealed marked vascular depletion and increased fibrosis in bone and surrounding soft tissues. Fibroblasts displayed characteristic radiation-induced nuclear enlargement and vesiculation. While most of the bone remained viable, focal areas of necrosis were present. Moreover, regions of otherwise living bone lacked an osteoblastic periosteal layer, accompanied by multifocal increases in osteoclastic resorption by multinucleated osteoclasts [35].

Bone undergoes continuous remodeling through a coordinated balance between osteoblast-driven formation and osteoclast-driven resorption, a process tightly regulated by hormonal and cytokine signaling. Disruption of this balance can lead to disorders marked by excessive bone loss and impaired regenerative capacity [36]. Radiation-induced microvascular damage results in hypocellular, hypovascular, and hypoxic bone tissue environments. However, the precise impact of radiation on bone remodeling remains a subject of debate. Ionizing radiation disrupts cellular function by generating ROS that exert direct inhibitory effects on cell proliferation and induce damage to osteoblasts, with DNA damage representing the most critical and well-characterized consequence [9, 37]. This may provide an acceptable explanation for the findings observed in the gamma-irradiated group.

The marked reduction in bone density observed in the ligature-induced periodontitis group (P) likely reflects the combined effects of bacterial biofilm accumulation and the consequent host inflammatory response, highlighting the pivotal role of inflammation-driven bone resorption in this model. The histopathological results were consistent with the radiographic observations, where untreated rats exhibited marked alveolar bone loss accompanied by pronounced disruption of the periodontal ligament architecture, and osteocytes exhibited enlarged lacunae, consistent with the expected inflammatory and osteolytic progression of the disease. In a recent study, rats’ teeth were ligated using 3 − 0, 4 − 0, or 5 − 0 silk sutures. Subsequently, ligated teeth exhibited significantly greater alveolar bone loss compared with the non-ligated control [14]. Similarly, in another study, ligature-induced periodontitis in a rat model produced marked alveolar bone resorption and prominent inflammatory responses within the periodontal tissues [38]. Consistent with our results, the ligature-induced periodontitis group demonstrated a statistically significant alveolar bone loss compared with the healthy control group [39, 40].

Osteocytes play a crucial role in initiating bone remodeling. Under persistent pathological and inflammatory stimuli within periodontal tissues, these cells are prone to senescence and subsequent dysfunction. This phenomenon, referred to as inflamm-aging, can disrupt normal bone remodeling dynamics and compromise overall bone homeostasis [41].

Osteoradionecrosis of the mandible occurs in approximately 5% of patients receiving radiotherapy for head and neck cancers. Current non-surgical management strategies for ORN demonstrate limited efficacy and are supported by a relatively small body of evidence [42]. Consequently, advancing our understanding of the underlying pathophysiology of ORN and exploring alternative therapeutic approaches is of critical importance. Alternative medicines and natural remedies have become more interesting for researchers owing to their low costs and reduced side effects [6].

Herbal preparations have long been employed worldwide as traditional therapeutic agents for a variety of human disorders, including those affecting the oral cavity and dentition [43]. Isoflavones, plant-derived phytoestrogens, exhibit remarkable estrogenic activity and play a fundamental role in bone physiology. Soy isoflavones contribute to osteoblastogenesis and differentiation, regulate osteoclastogenesis and osteoclast mineralization, and influence the differentiation of bone marrow mesenchymal stromal cells. They also support calcium homeostasis by modulating extracellular calcium and vitamin D levels. Additionally, soy isoflavones alleviate oxidative stress within the endoplasmic reticulum and mitochondria, thereby influencing cellular senescence, autophagy, and bone remodeling processes [44]. Collectively, these properties highlight the therapeutic potential of soy-isoflavones in osteoporosis management through the enhancement of bone health, regulation of metabolic processes, and diminution of oxidative stress.

Previous studies have investigated the effects of soy isoflavones on bone metabolism and their potential to support bone function. However, the therapeutic potential of soy isoflavones in mitigating gamma radiation-induced damage to alveolar bone remains poorly investigated. This concept formed the basis of the present study, prompting the question of whether isoflavones could be employed to mitigate the detrimental effects of gamma radiation on alveolar bone. The doses of soy-isoflavones employed in the present study (20 and 50 mg/kg) fall within the range commonly reported in preclinical studies investigating the radioprotective and anti-inflammatory properties of soy-isoflavones [14, 16, 45]. Previous experimental evidence has demonstrated that these doses exert biologically relevant protective effects against radiation-induced tissue injury while maintaining a favorable safety profile in animal models [16, 46].

Radiographically, both isoflavone doses (20 and 50 mg/kg) produced significant improvements in bone density relative to the untreated groups. The greatest mean bone density was observed in the P50 group, followed by the R50 group, demonstrating a stronger osteoprotective response at the higher dose. This may indicate that isoflavone may enhance bone regeneration and help counteract bone loss associated with radiation- or ligature-induced periodontitis.

Treatment with isoflavone (R20 and R50) enhanced Haversian canal vascularity and improved osteocyte organization, with the higher dose producing significant histological improvement. These outcomes indicate that isoflavone supports post-irradiation bone repair and revascularization, which are key processes for maintaining skeletal homeostasis. Likewise, isoflavone administration (P20 and P50) promoted periodontal fiber re-establishment and improved osteocyte organization at higher doses. Exceptionally, the P50 group exhibited significant recovery of the alveolar crest and proper periodontal ligament alignment, accompanied by restoration of Haversian and Volkmann’s canals, reflecting enhanced vascularization and bone remodeling. Overall, these results align with the established roles of isoflavones in modulating osteoblast activity, stimulating collagen synthesis, and promoting angiogenesis.

Our findings are consistent with previous reports indicating that soy-isoflavones enhance collagen deposition and stimulate the expression of genes involved in osteogenesis and angiogenesis [16, 47]. Epidemiological evidence suggests a positive association between higher isoflavone consumption and increased bone mineral density. This beneficial effect is attributed to the favorable pharmacokinetic characteristics of isoflavones, particularly their efficient absorption and distribution to target tissues. Additionally, isoflavones such as genistein and daidzein have been shown to exert immunomodulatory effects in humans [7].

Among the treated groups (R20, P20, R50, and P50), bone density was the lowest in the R20 group, followed by P20 and R50, with the highest values observed in P50. This pattern may reflect the ability of soy-isoflavones to attenuate bone loss in rats, particularly at higher doses [48]. A recent in vivo investigation reported that dietary supplementation with soy-isoflavones at 352 mg/g effectively prevented high-fat-diet–induced declines in bone mass, preserving bone volume, trabecular number, density, and mechanical strength [48]. As well, soy isoflavones alleviated periodontitis and reduced alveolar bone loss in ovariectomized rats [49].

Soy isoflavones markedly attenuated the ligature-induced reductions in alveolar bone height and bone volume fraction in rats. It also ameliorated the adverse microarchitectural changes in trabecular bone caused by ligature placement, preserving parameters such as trabecular thickness and bone mineral density [50]. Daily administration of soy-isoflavones markedly attenuated ligature-induced alveolar bone loss in rats. Treatment with soy-isoflavones also significantly restored the ligature-induced reduction in bone volume fraction. Moreover, it effectively reversed the deterioration of trabecular bone microarchitecture, improving parameters such as trabecular thickness, bone mineral density, trabecular separation, and structure model index [51].

The differential response to soy-isoflavones observed between the radiation- and ligature-induced models may be attributed to their distinct pathophysiological mechanisms. Radiation-induced bone damage is primarily mediated by microvascular compromise, depletion of cellular components, and enhanced oxidative stress [9, 37]. In contrast, ligature-induced periodontitis is predominantly driven by bacterial biofilm accumulation and the subsequent host inflammatory response [14, 38–40]. These mechanistic differences may modulate the extent to which soy isoflavones exert their protective effects on bone remodeling and inflammatory pathways.

The more pronounced response observed in the ligation groups, compared with the radiation groups, may be attributed to the predominantly inflammatory mechanism underlying ligation-induced bone loss. In contrast, radiation-induced damage involves direct cellular injury and compromised osteogenesis in addition to inflammation, which may collectively limit the overall protective efficacy of isoflavones.

Isoflavones contribute to calcium metabolism by promoting the mobilization of calcium from bone into the circulation, thereby enhancing serum calcium levels. Although their mechanisms of action are not yet fully elucidated, current evidence suggests that isoflavones exert dual effects on bone remodeling as they suppress bone resorption and simultaneously promote bone formation via stimulating osteoblastic proliferation and differentiation, inhibiting osteoclastic resorption, and modulating bone turnover markers [52]. Isoflavones enhance bone formation mainly by activating estrogen receptors on osteoblasts, functioning as phytoestrogens due to their structural similarity to endogenous estrogens [51, 52]. Moreover, isoflavones exhibit remarkable antioxidant and anti-inflammatory properties, helping to alleviate oxidative stress and suppress the release of pro-inflammatory cytokines [51].

Overall, the observed improvements in both bone density and histological architecture suggest a potential beneficial effect of isoflavone in mitigating gamma radiation- and ligature-induced periodontitis, although the presence of overlapping statistical groupings indicates that these changes were not consistently distinct across treatment conditions. Notably, the higher dose (50 mg/kg) produced more pronounced effects, which may be attributed to the estrogen receptor-mediated osteogenic effects, along with anti-inflammatory and antioxidant actions. Accordingly, the null hypothesis was rejected.

Although the present study provides valuable understandings of the therapeutic effects of isoflavone against gamma radiation- and ligature-induced periodontitis, several limitations should be acknowledged when interpreting the conclusions. First, the study was limited by a relatively short experimental duration and assessment at a single time point. This may not fully reflect the long-term impact of isoflavone on bone remodeling dynamics or periodontal tissue regeneration. Second, only two doses of isoflavone (20 and 50 mg/kg) were evaluated. While both demonstrated therapeutic benefits, further dose-response investigations are required to define the optimal therapeutic range to achieve maximal bone-protective and regenerative benefits, and to evaluate potential toxicity effects at higher doses. Third, the study did not include molecular, biochemical, or systemic outcome analyses. Consequently, the underlying mechanisms, such as the regulation of osteogenic pathways, oxidative stress responses, and inflammatory mediators, were not clarified. Fourth, the histological analysis was limited to qualitative descriptive assessment without morphometric quantification or standardized scoring systems, which may reduce the precision and statistical strength of the findings. Fifth, the exclusive use of male rats, intended to reduce hormonal variability, restricts the generalizability of the results. Given the phytoestrogenic properties of soy isoflavone, inclusion of both sexes in future work is essential to assess possible sex-specific differences. Finally, although the study relied on radiographic and histological evaluations, the addition of functional assessments such as micro-CT imaging or periodontal attachment level measurement would further enhance the translational relevance of the study.

In light of the present findings, several directions for future research are recommended. Long-term studies incorporating dose-response assessments are needed to validate the durability and safety profile of isoflavones. Molecular analyses should also be undertaken to clarify the signaling pathways supporting its osteoprotective and tissue-regenerative effects. Moreover, future studies should include quantitative morphometric analysis or standardized scoring systems. Comparative investigations between isoflavone and conventional therapeutic modalities, such as antioxidants and anti-inflammatory agents, may help identify potential synergistic interactions. In addition, well-designed clinical trials are necessary to evaluate the translational applicability of isoflavone as an adjunctive therapy for patients receiving head and neck RT or those with chronic periodontitis.

## Conclusion

Within the limitations of this study, isoflavone demonstrated a significant therapeutic effect against gamma radiation- and ligature-induced periodontitis in male albino rats. The data indicate that isoflavone, particularly at a dose of 50 mg/kg body weight, can preserve bone architecture, enhance vascularity, and promote periodontal regeneration, making it a promising adjunctive agent in the prevention and management of radiation-associated and inflammatory bone loss.

## Data Availability

All data generated or analyzed during this study are included in this published article.
